# Are Bogs Reservoirs for Emerging Disease Vectors? Evaluation of *Culicoides* Populations in the Hautes Fagnes Nature Reserve (Belgium)

**DOI:** 10.1371/journal.pone.0066893

**Published:** 2013-06-14

**Authors:** Jean-Yves Zimmer, François Smeets, Grégory Simonon, Jean Fagot, Eric Haubruge, Frédéric Francis, Bertrand Losson

**Affiliations:** 1 Unit of Functional and Evolutionary Entomology, Department of Agronomic Sciences, Gembloux Agro-Bio Tech, University of Liege, Gembloux, Belgium; 2 Laboratory of Parasitology and Parasitic Diseases, Department of Infectious and Parasitic Diseases, Faculty of Veterinary Medicine, University of Liege, Liege, Belgium; Thomas Jefferson University, United States of America

## Abstract

Several species of *Culicoides* (Diptera: Ceratopogonidae) biting midges serve as biological vectors for the bluetongue virus (BTV) and the recently described Schmallenberg virus (SBV) in northern Europe. Since their recent emergence in this part of the continent, these diseases have caused considerable economic losses to the sheep and cattle industries. Much data is now available that describe the distribution, population dynamics, and feeding habits of these insects. However, little is known regarding the presence of *Culicoides* in unusual habitats such as peaty marshes, nor their potential vector capacity. This study evaluated *Culicoides* biting midges present in the bogs of a Belgian nature reserve compared to those residing at a nearby cattle farm. *Culicoides* were trapped in 2011 at four different sites (broadleaved and coniferous forested areas, open environments, and at a scientific station) located in the Hautes Fagnes Nature Reserve (Belgium). An additional light trap was operated on a nearby cattle farm. Very high numbers of biting midges were captured in the marshy area and most of them (70 to 95%) were *Culicoides impunctatus*, a potential vector of BTV and other pathogens. In addition, fewer numbers of C. *obsoletus/C. scoticus* species, *C. chiopterus*, and *C. dewulfi* were observed in the bogs compared to the farm. The wet environment and oligotrophic nature of the soil were probably responsible for these changes in the respective populations. A total of 297,808 *Culicoides* midges belonging to 27 species were identified during this study and 3 of these species (*C. sphagnumensis*, *C. clintoni* and *C. comosioculatus*) were described in Belgium for the first time.

## Introduction

Numerous insects worldwide serve as vectors for various human and animal diseases and hematophagous diptera represent an important group. Included in this group are the biting midges that belong to the family Ceratopogonidae, genus *Culicoides* Latreille 1809 [1]. About 1,400 species of *Culicoides* (mainly crepuscular or nocturnal) are described throughout the world, but only a few species may act as biological virus vectors [2], [3], [4], [5], [6], [7], [8], [9], [10], [11]. In recent years these bloodsucking insects (ranging between 1–4 mm) have been associated with outbreaks of important viral epizoonoses in northern Europe, including bluetongue (BT) in 2006 [12] and Schmallenberg disease (SB) in 2011 [13], [14]. Bluetongue is an infectious, non-contagious disease which affects domestic and wild ruminants. Since the emergence of BT serotype 8 in northern Europe in August 2006 [12], considerable direct and indirect economic losses have been recorded in the sheep and cattle industries over a large part of the European Union [15]. Analysis of the evolution of BT in Europe highlights the risk of the emergence of new serotypes or viral recombination when multiple serotypes are present in the same region, such as in France [15].

Biting midges are also a source of nuisance through the bites of females. Their presence can therefore hinder the economic development of some regions, hampering agricultural and forestry activities as well as tourism development [16].

Due to the economic impact of these diseases, most adult midge trapping campaigns have previously been conducted on farms utilizing different types of light traps. In Belgium and neighbouring countries, entomological monitoring programs were conducted between 2007 and 2012 by the Federal Agency for the Safety of the Food Chain. Larval development of such midges happens preferentially in the uppermost layer of semi-aquatic or wet substrates, which are rich in organic debris [17]. Wetlands such as bogs and marshes have a high biological value and are often included in national or international protection programs. However these habitats could represent highly suitable biotopes with the capacity of sustaining the development of the immature biting midge stage. Additionally, the original introduction of BT virus (BTV) to Belgium probably took place during the spring of 2006 and not far from the Hautes Fagnes Nature Reserve [18] and a flock of sheep grazing in 2007 in this area was infected with BTV. Despite these factors, bogs to date have been insufficiently studied with respect their ability to sustain the growth and development of bloodsucking midges. Consequently a targeted surveillance in this reserve, which corresponds to a destination area for many migrating birds, is therefore required.

The objectives of the present study were to characterize the biting midge populations present in different biotopes of the Hautes Fagnes Nature Reserve (Belgium) and to compare these midges to those captured on a nearby cattle farm.

## Materials and Methods

### Study Sites

This study was conducted in 2011 in a wetland in the province of Liège (eastern Belgium), located at the edge of the Hautes Fagnes Nature Reserve (50° 30' to 50° 33' N; 6° 4' to 6° 7' E) and on a cattle farm located in Jalhay in the same province (50° 34' N; 5° 55' E) approximately 13 km from the wetland. The Hautes Fagnes Nature Reserve is a high plateau characterized by acidic and oxygen-free substrates situated at an altitude of over 500 m above sea level. Several streams originate from the peaty moorlands of this reserve that covers an area of about 5,000 ha. The climatic, geological, and orographic conditions of the reserve are well defined [19] and can sustain the development of many rare plant and animal species. Each year, a flock of approximately 1,900 rustic sheep are put to pasture at the Hautes Fagnes Nature Reserve between early May and late September.

Four study sites were selected within the wetland, namely a broadleaved forest area, a coniferous forest area, an open area (corresponding to a peaty moor highly exposed to wind) with a pond located at about 100 m from the meadow where part of the sheep flock grazes, and the Scientific Station of Hautes Fagnes (SSHF) which is located at Mount Rigi in the reserve approximately 5 km from the previous three study sites.

### Ethics Statement

A permit of access and circulation within the study sites of the Hautes Fagnes Nature Reserve was issued by the Cantonment of Verviers, Department of Nature and Forests, Walloon Public Service. A permission allowing the realization of light trapping in this reserve was also granted by the Department of Nature and Forests, Directorate General for Natural Resources and Environment, Walloon Public Service. A permission of access and realization of light trapping on the cattle farm located in Jalhay was finally given by the farmer. No protected species were sampled during this study.

### *Culicoides* Collection

In order to monitor the *Culicoides* biting midge populations, an “OVI” (Onderstepoort Veterinary Institute) type UV light trap was operated at each of the five study sites (four sites selected within the wetland and the nearby cattle farm) for 24 h (at the SSHF) or 48 h (at the four other sites) once a week between May 26 and September 28, 2011. The traps, which work on the basis of light attraction, were powered by 12 V batteries (at the cattle farm, broadleaved, coniferous, and open areas) or connected directly to the 220 V mains at the SSHF. A nylon wire mesh with an aperture of 5 mm was added to the trap to facilitate the trapping of tiny insects. Traps were suspended so as to position a collecting flask containing approximately 400 ml of a mixture of water and detergent at about 1.5 m above the ground. The traps located in the broadleaved, coniferous, and open areas were approximately 400 m from each other. The trap at the SSHF was located at the edge of an open area where sheep graze while the trap at the farm was operated a few meters from a herd of calves and a silo of beet pulp.

### *Culicoides* Identification

Collected samples were first filtered through an iron wire mesh with an aperture of 0.5 mm and transferred into a solution of 80% ethanol. Trapped insects were then sorted by genus using a stereomicroscope (10–40× magnification) in order to highlight *Culicoides* biting midges. Respective specimens were then sexed, counted, and classified to species level using a morphological key [20]. However, *C. obsoletus* (Meigen) and *C. scoticus* Downes & Kettle females could not be morphologically distinguished and were therefore classified as the *Obsoletus* complex. The nutritional status of females (non-pigmented, pigmented, gravid, and engorged) based on abdominal pigmentation [21] was also recorded, taking into account the recommendation of Harrup et al. [22].

## Results

The total number of traps varied between 14 (SSHF and cattle farm sites) and 16 (broadleaved, coniferous, and open areas) due to damage caused by rodents to the equipment. During this study a total of 297,808 Culicoides biting midges were captured. Numbers of midges collected by site were as follows: SSHF (n = 213,120), open area (n = 32,727), cattle farm (n = 23,667), coniferous area (n = 15,340), and the broadleaved area (n = 12,954). At the SSHF, an impressive capture of 179,426 *Culicoides* was recorded on July 9. *Culicoides* species heterogeneity varied between study sites, ranging between 16 and 21 as follows: SSHF (21 species), open area and cattle farm (18 species each), coniferous area (17 species), and broadleaved area (16 species). Seven species represented more than 99% of the total catches at each study site. The species composition of the five study sites is presented in Table 1. At the four study sites located in the reserve or at its periphery 70–95% of biting midges belonged to the species *C. impunctatus* Goetghebuer (which was nearly absent on the nearby cattle farm) whereas less than 10% of specimens belonged to the *Obsoletus* complex that comprised the two most abundant species at the nearby farm. *Culicoides chiopterus* (Meigen) and *C. dewulfi* Goetghebuer were poorly represented on the wetland sites and *C. albicans* (Winnertz) was not observed on the farm but was well represented in the coniferous and open areas.

**Table 1:** *Culicoides* species composition (number (percentage)) trapped at the five study sites.

*Culicoides* species	Coniferous forest area	Broadleaved forest area	Open area	Scientific station	Cattle farm
*C. achrayi*	1 (<0.1)	0 (0)	1 (<0.1)	23 (<0.1)	22 (<0.1)
*C. albicans*	2,072 (13.5)	81 (0.6)	9,432 (28.8)	27 (<0.1)	0 (0)
*C. albihalteratus*	2 (<0.1)	4 (<0.1)	74 (0.2)	6 (<0.1)	0 (0)
*C. chiopterus*	7 (<0.1)	2 (<0.1)	1 (<0.1)	4 (<0.1)	545 (2.3)
*C. clintoni*	5 (<0.1)	7 (<0.1)	12 (<0.1)	4 (<0.1)	0 (0)
*C. comosioculatus*	0 (0)	0 (0)	0 (0)	12 (<0.1)	0 (0)
*C. deltus*	2 (<0.1)	8 (<0.1)	1 (<0.1)	0 (0)	1 (<0.1)
*C. dewulfi*	0 (0)	4 (<0.1)	2 (<0.1)	6 (<0.1)	2,845 (12.0)
*C. fascipennis*	3 (<0.1)	3 (<0.1)	2 (<0.1)	13 (<0.1)	1 (<0.1)
*C. festivipennis*	0 (0)	0 (0)	0 (0)	2 (<0.1)	5 (<0.1)
*C. furcillatus*	0 (0)	0 (0)	0 (0)	2 (<0.1)	4 (<0.1)
*C. grisescens*	19 (0.1)	41 (0.3)	55 (0.2)	426 (0.2)	1 (<0.1)
*C. heliophilus*	0 (0)	0 (0)	3 (<0.1)	0 (0)	0 (0)
*C. impunctatus*	12,604 (82.2)	11,461 (88.5)	23,089 (70.6)	202,664 (95.1)	13 (<0.1)
*C. kibunensis*	0 (0)	0 (0)	0 (0)	21 (<0.1)	16 (<0.1)
*C. lupicaris*	0 (0)	0 (0)	0 (0)	0 (0)	6 (<0.1)
*C. minutissimus*	0 (0)	0 (0)	4 (<0.1)	0 (0)	0 (0)
*C. nubeculosus*	0 (0)	0 (0)	0 (0)	0 (0)	3 (<0.1)
*C. obsoletus/C. scoticus*	545 (3.6)	1,296 (10.0)	35 (0.1)	9,389 (4.4)	19,391 (81.9)
*C. pulicaris*	46 (0.3)	23 (0.2)	8 (<0.1)	154 (<0.1)	721 (3.0)
*C. punctatus*	2 (<0.1)	8 (<0.1)	3 (<0.1)	59 (<0.1)	91 (0.4)
*C. segnis*	3 (<0.1)	11 (<0.1)	1 (<0.1)	19 (<0.1)	0 (0)
*C. sphagnumensis*	21 (0.1)	4 (<0.1)	4 (<0.1)	286 (0.1)	0 (0)
*C. stigma*	0 (0)	0 (0)	0 (0)	0 (0)	1 (<0.1)
*C. subfascipennis*	1 (<0.1)	0 (0)	0 (0)	1 (<0.1)	1 (<0.1)
*C. truncorum*	7 (<0.1)	1 (<0.1)	0 (0)	2 (<0.1)	0 (0)
TOTAL	15,340	12,954	32,727	213,120	23,667

This study identified *C. chiopterus*, *C. fascipennis* (Staeger), *C. grisescens* Edwards, *C. impunctatus*, *C. obsoletus/C. scoticus, C. pulicaris* (L.) and *C. punctatus* (Meigen) at all five study sites. In contrast, *C. albicans, C. albihalteratus* Goetghebuer, *C. clintoni* Boorman, *C. segnis* Campbell & Pelham-Clinton and *C. sphagnumensis* Williams were observed at the four wetland sites but not at the farm. *Culicoides truncorum* Edwards was identified at some of the wetland sites while *C. lupicaris* Downes & Kettle, *C. nubeculosus* (Meigen) and *C. stigma* (Meigen) were observed only at the farm. *Culicoides heliophilus* Edwards and *C. minutissimus* (Zetterstedt) were only observed in the open area and *C. comosioculatus* Tokunaga only at the SSHF. *Culicoides achrayi* Kettle & Lawson, *C. dewulfi*, *C. festivipennis* Kieffer, *C. furcillatus* Callot, Kremer & Paradis, *C. kibunensis* Tokunaga, *C. subfascipennis* Kieffer, and *C. deltus* Edwards were observed in some wetland sites and at the farm. A total of 27 *Culicoides* species were identified during the study period. Females greatly predominated, accounting for approximately 93.5% of *Culicoides* in both woody areas, and about 99.5% of catches taken in the open area, the SSHF, and the cattle farm.

The nutritional status of respective females is described in Table 2. Pigmented females were particularly abundant at the SSHF, as well as in both woody areas. Gravid females were particularly numerous in the open environment, while non-pigmented females were abundant within the farm. Blood-engorged females were poorly represented at all study sites.

**Table 2:** Nutritional status (percentage) of female *Culicoides* trapped at the five study sites.

Nutritional status	Coniferous forest area	Broadleaved forest area	Open area	Scientific station	Cattle farm
Non-pigmented	4.1	11.3	1.7	3.2	46.0
Pigmented	68.0	83.5	13.4	95.7	36.0
Blood-engorged	<0.1	<0.1	<0.1	<0.1	/
Gravid	27.8	5.1	84.9	1.1	18.0

## Discussion

This study confirmed the presence of significant populations of adult *Culicoides* in the Hautes Fagnes Nature Reserve. This was predictable given the death of approximately 130 sheep grazing in this area in 2007 during the BTV epizoonosis. This area is characterized by its marshy nature and the presence of wet substrates rich in organic material.

Even if the light trapping surveillance conducted during this study did not accurately reflect the *Culicoides* biting midge population present [23], this method is widely used since it easily allows comparisons between sites and the identification of the types of midges at different locations to be carried out. The *Culicoides* species richness observed for the cattle farm (18 species) was high compared to species variability described by other studies conducted in Belgium or in other northern European farms. For example, a total of 17 species were captured in a Belgian meadow compared to only 10 species on a nearby cattle farm [24]. In northern France, 10 species were observed outside the animal accommodation buildings, compared to 7 species identified indoors [25]. This greater species diversity could be explained by the proximity of the wetland, characterized by bogs that may function as breeding sites for several midge species. The most abundant *Culicoides* species observed at the farm paralleled the species observed on most other cattle farms in northern Europe with the majority of species belonging to the *Obsoletus* complex, as well as *C. dewulfi*, *C. pulicaris,* and *C. chiopterus* [25], [26], [27], [28]. Note that the number of specimens of this last species is likely underestimated as demonstrated by Carpenter et al. [23] for light trapping surveys. Thirteen other minor species that are not often found on farms were identified in the present study, particularly *C. impunctatus*.

This species is considered to be a potential vector of BTV since *C. impunctatus* is able to support virus multiplication in the laboratory after its ingestion [29]. Moreover, this species is probably an important natural vector of *Haemoproteus* spp., parasites of passerine birds in Europe [30]. However, *C. impunctatus* females are autogenous, not requiring a blood meal to lay their first batch of eggs [31], [32]. This species is furthermore a generalist feeder that prefers large mammals and is known to attack humans opportunistically [33], [34], [35]. These characteristics may limit its efficiency as a vector, but autogeny allows this species to reach huge population densities even if hosts are scarce [36]. *Culicoides impunctatus* seems very common in bogs [37] where the larvae feed on oligotrophic soils [38]. In Scotland, this species is a major pest and the dominant species living in bogs where the larvae develop in wet soil on slopes that ensures a constant flow of water, as well as in neighbouring acid meadows [39]. Wetter areas of these bogs are preferentially colonized by *C. albicans* and *C. heliophilus*, whereas very wet areas support *C. truncorum* populations. Acid meadows located at greater distances from these bogs sustain the growth of *C. kibunensis* and *C. obsoletus* [39]. Moreover, these species also favour different soil depths. *Culicoides impunctatus*, *C. pallidicornis* Kieffer, and *C. obsoletus* larvae live at the surface layer of their breeding site (in the top 2.5 cm), *C. kibunensis* at medium depths, while *C. albicans* and *C. heliophilus* are found deeper (below 5 cm) [38], [40]. These different species occupy different niches in a common site and so can probably coexist without competition for nutrients. These elements might therefore explain the abundance of *C. impunctatus* at the different study sites of the Hautes Fagnes Nature Reserve.

The two species forming the *Obsoletus* complex as well as *C. dewulfi* and *C. chiopterus* seem to prefer anthropogenic substrates or substrates related to livestock as breeding sites [41], which may explain why these two species were more commonly found at the farm. This is particularly true for *C. obsoletus* which breeds in manure [42] and is the most abundant species isolated from Belgian farms as well as in maize and other types of silage residues [41], [43]. Used bovine litter adhering to the walls of stables seems also to support the larval development of this species [44]. However, members of the *Obsoletus* complex also frequent the wet litter of many boreal forests [45], stagnant water reservoirs, and marshes with dense vegetation [46], as well as compost heaps of leaves and tree holes [47]. The larval stages of *C. chiopterus* and *C. dewulfi* prefer cattle and horse dung found in meadows [38], [41].

The abundance of female *Culicoides* compared to males throughout this study could be partly explained by a lower attraction of males to light or perhaps by a difference in the diet between the sexes, that is, males are flower-dwelling, probably preferentially frequenting vegetation and tree tops that may explain why few were captured in the light traps used. Bloodsucking females are more likely to be found at ground level near livestock and therefore more likely to be near the UV traps placed at a level of 1.5 m. These two assumptions remain valid by observing that a Rothamsted suction trap operating at a height of 12 m indeed recorded a far greater proportion of *Culicoides* males (23.5%) [26]. However the fact that *C. impunctatus* males do not fly far from their emergence site [32] could also explain the low number of males trapped.

The predominance of non-pigmented females (newly hatched females having not yet had a blood meal) on the farm could be explained by the presence of breeding sites in the immediate surroundings of the trap or by the active search of a host in order to take a first blood meal. Remember that the real number of newly emerged females is probably underestimated [22]. The abundance of gravid females at the open site could be explained by the search for egg laying sites within this particularly wet environment that includes a pond. There is indeed a relationship between the proximity of traps relative to breeding sites and the nutritional status of captured females [26], [48].

Finally, the study allowed for the identification of three *Culicoides* species not previously recorded in Belgium (identification confirmed by Professor Delécolle, Institute of Parasitology and Tropical Pathology, University of Strasbourg): *C. sphagnumensis* (n = 315), *C. clintoni* (n = 28), and *C. comosioculatus* (n = 12). Consequently the number of *Culicoides* species identified in Belgium is now 52. Three other species (*C. albicans*, *C. fascipennis* and *C. grisescens*) are quite rare on Belgian farms were also regularly observed in the Hautes Fagnes Nature Reserve.

## Conclusions

The protected wetlands of northern Europe, characterized by bogs and considered in some countries (including Belgium) to be strict nature reserves for flora and fauna, harbour important populations of *Culicoides*. Bogs may thus constitute a reservoir for the development of *Culicoides* biting midges, such as *C. impunctatus*, considered to be potential vectors for BTV and other pathogens. Particular characteristics of the wet micro-habitats constituting these areas could indeed explain the abundance of species infrequently encountered on farms, especially *C. impunctatus* and *C. albicans*. Protected wetlands do not therefore seem responsible for the *Culicoides* populations observed in Belgian farms, even if the proximity of bogs slightly influenced the *Culicoides* species composition on these farms.
